# Outcomes for Women With Diabetes at Late Preterm Gestation Who Received Antenatal Corticosteroids: A Population‐Based Cohort Study

**DOI:** 10.1111/ajo.70164

**Published:** 2026-07-28

**Authors:** Ibinabo Ibiebele, Kata Kraljevic, Sarah Glastras, Tessa Weir, Deborah Randall, Tanya Nippita

**Affiliations:** ^1^ Reproduction and Perinatal Centre – Northern Precinct, Faculty of Medicine and Health, the University of Sydney Sydney New South Wales Australia; ^2^ Kolling Institute, University of Sydney & Northern Sydney Local Health District St Leonards New South Wales Australia; ^3^ Department of Obstetrics and Gynaecology Royal North Shore Hospital, North Sydney Local Health District St Leonards New South Wales Australia; ^4^ Department of Diabetes Endocrinology and Metabolism, Royal North Shore Hospital, North Sydney Local Health District Sydney New South Wales Australia; ^5^ Department of Endocrinology Nepean Blue Mountains Hospital, Nepean Blue Mountains Local Health District Kingswood New South Wales Australia; ^6^ Clinical Excellence Commission, New South Wales Health St Leonards New South Wales Australia

**Keywords:** antenatal corticosteroids, diabetes, neonatal hypoglycaemia, neonatal intensive care unit, preterm birth

## Abstract

**Background:**

The use of antenatal corticosteroids appears to be increasing despite limited and conflicting evidence about its efficacy among women with diabetes in pregnancy.

**Aim:**

To separately assess the effects of maternal diabetes and the effects of antenatal corticosteroid administration prior to late preterm birth on adverse maternal and neonatal outcomes.

**Materials and Methods:**

Cohort study of women who gave birth ≥ 35 weeks gestation between September 2016 and March 2021 in two local health districts in New South Wales, Australia. Women were grouped into one of the following: (1) diabetes and received corticosteroids, (2) diabetes and no corticosteroids, (3) no diabetes and received corticosteroids. Modified Poisson regression was used to estimate adjusted relative risks (aRR).

**Results:**

A total of 5 067 women were included (Group 1 = 205, Group 2 = 4 028, Group 3 = 834). Women who received corticosteroids were characterised by higher proportions of hypertension, hospital‐based medical antenatal care, multiple births and caesarean section. There were no differences in maternal and neonatal outcomes for women who had diabetes and received corticosteroids compared to those who had diabetes and did not receive corticosteroids. Women with diabetes who received corticosteroids had a higher risk of infants with neonatal hypoglycaemia (aRR 1.61, 95% CI 1.28–2.02) and admission to nursery or intensive care unit (aRR 1.18, 95% CI 1.02–1.38), compared to women without diabetes who received corticosteroids.

**Conclusions:**

There is limited evidence of benefit for the use of antenatal corticosteroids in late preterm gestations, regardless of diabetes status. Further studies are required to identify potential longer‐term benefits or harms.

## 1 Introduction

Preterm birth, defined as delivery before 37 weeks of gestation, remains a significant challenge in obstetric care, and it is a cause of significant neonatal morbidity and mortality. Late preterm birth, classified as birth between 34^+0^ and 36^+6^ weeks, accounts for 69% of all preterm births in Australia and carries increased risks of respiratory and neurodevelopmental complications compared to term births [1]. While the absolute risks are low, this equates to substantial morbidity given the number of late preterm births.

The prevalence of pre‐gestational and gestational diabetes is increasing in Australia and internationally. Diabetes during pregnancy is associated with neonatal hypoglycaemia, delayed surfactant synthesis and increased rates of caesarean section, with the latter independently associated with neonatal respiratory morbidity including respiratory distress syndrome and transient tachypnoea of the newborn [2, 3].

One of the most impactful interventions for mitigating preterm birth risk is the administration of antenatal corticosteroids to enhance lung maturation and surfactant production. In uncomplicated pregnancies up to 34 weeks gestation, antenatal corticosteroids have been shown to reduce the incidence of respiratory distress syndrome, cerebrovascular haemorrhage, necrotising enterocolitis, sepsis and neonatal death [4]. In late preterm gestations, previous studies suggest that antenatal corticosteroids reduce the incidence of stillbirth, neonatal death and need for respiratory support [5, 6, 7, 8]. However, studies examining the efficacy of corticosteroids have either excluded women with diabetes [7, 8] or included a very small population of women with diabetes such that the findings are not generalisable to a wider population of women with diabetes [5, 6]. Concern about neonatal hypoglycaemia, maternal hyperglycaemia and appropriate titration of insulin are the major reasons for exclusion of women with diabetes. Specifically, corticosteroids are known to transiently elevate maternal blood glucose levels [9], which could complicate glycaemic management and impact both maternal and neonatal health outcomes.

The use of antenatal corticosteroids among women with diabetes is increasing despite limited and contradictory evidence about its efficacy [10, 11, 12]. Our study aims to examine the association between antenatal corticosteroids and adverse maternal and neonatal outcomes among women with and without diabetes birthing from 35^+0^ weeks gestation to separately assess the effects of corticosteroids and diabetes on maternal and neonatal outcomes.

## 2 Methods

This cohort study included women who gave birth at 35^+0^ weeks gestation onwards between September 2016 and March 2021, at public maternity facilities within the Northern Sydney and Central Coast Local Health Districts (LHDs) of New South Wales, Australia. Northern Sydney LHD is located within metropolitan Sydney, covers an area of 900 km^2^ with a population of over 980 000 residents [13]. Central Coast LHD is located just north of Sydney with an area of 1853 km^2^ and a population of 350 000 residents [13]. In 2021, there were over 13 900 births across both LHDs. The study utilised routinely collected maternity data from the eMaternity clinical database, which captures sociodemographic, medical, obstetric and birth information for all births of at least 20 weeks gestation or 400 g birthweight. Information is entered into the database by midwives at antenatal visits and during the birth admission, and only de‐identified data were provided to the researchers.

The exposures of interest were diagnosis of diabetes mellitus and whether antenatal corticosteroids were administered prior to birth. Diabetes included both pre‐gestational (Type 1, Type 2) and diabetes diagnosed during the pregnancy (gestational diabetes). The diagnosis of gestational diabetes was made according to International Association of the Diabetes and Pregnancy Study Group (IADPSG) criteria [14]. Administration of antenatal corticosteroids included incomplete and complete courses. A complete course was defined as 2 doses given either 12 or 24 h apart, and more than 24 h and less than 8 days before birth. An incomplete course of corticosteroids was defined as at least one dose given less than 24 h before birth. A rescue dose may have been given a week or more after the completed course. For pregnancies of < 34^+6^ weeks gestation with imminent preterm birth or birth predicted within 7 days, administration of antenatal corticosteroids is recommended. However, beyond this clinical scenario, the decision regarding administration of antenatal corticosteroids is at the discretion of the clinician. For women with diabetes in pregnancy, administration of antenatal corticosteroids is at the discretion of the individual obstetrician, and management of the glucose level in response to antenatal corticosteroid administration is under the supervision of the endocrinologist. Insulin infusions were not routinely used after antenatal corticosteroid administration. Women were assigned to one of three groups: (1) women who had a diagnosis of diabetes and received antenatal corticosteroids prior to delivery (study group), (2) women who had a diagnosis of diabetes but did not receive antenatal corticosteroids (comparison group), and (3) women who did not have a diagnosis of diabetes and received antenatal corticosteroids (comparison group). Women who did not have diabetes nor receive antenatal corticosteroids were excluded.

Maternal outcomes included postpartum haemorrhage and maternal infection. Postpartum haemorrhage was derived from volume of blood loss in the first 24 h (primary) and after 24 h (secondary) since the birth and was defined three ways: ≥ 500 mL total, ≥ 500 mL primary, and ≥ 1 000 mL total blood loss. Neonatal outcomes included jaundice, hypoglycaemia (blood glucose < 2.6 mmol/L), respiratory distress (difficulty breathing that persists longer than 4 h and radiologic findings of increased density in both lungs with a ‘ground glass’ appearance), admission to neonatal intensive care unit (NICU) or special care nursery (SCN), stillbirth (fetal death ≥ 35 weeks gestation) and neonatal death (death of a live born infant within 28 days of birth) during the birth admission.

Other variables of interest included maternal age, body mass index (BMI), maternal self‐reported country of birth grouped into regions according to the Australian Bureau of Statistics (ABS) Standard Australian Classification of Countries, smoking, socioeconomic status, remoteness, parity, plurality, type of diabetes (gestational diabetes, Type 1, Type 2), diabetes management (diet only, oral hypoglycaemics, insulin), hypertension, previous caesarean delivery, model of care, mode of delivery, gestational age at birth, small‐for‐gestational age (SGA, birthweight < 10th percentile) [15, 16], large‐for‐gestational age (LGA, birthweight > 90th percentile) [15, 16], and baby's year of birth. BMI was calculated using self‐reported pre‐pregnancy weight and maternal height recorded at the booking visit. Socioeconomic status and remoteness were area‐level measures based on maternal residential postcode mapped to the ABS Index of Relative Disadvantage and grouped into population quintiles, and the ABS Statistical Geography Standard Remoteness Area Structure, respectively.

### 2.1 Statistical Analysis

Frequencies and proportions, means and standard deviation were used to describe the characteristics of the study population. Characteristics of the study groups were compared using chi‐square tests for categorical data and *t*‐tests for continuous data. Outcomes were compared according to the presence or absence of antenatal corticosteroids (Group 1 vs. Group 2), and the presence or absence of diabetes (Group 1 vs. Group 3). Modified Poisson regression with robust error variances was used to estimate unadjusted and adjusted relative risks and their 95% confidence intervals. For multivariable regression models, potential confounders were selected based on a priori knowledge from clinical experience and univariate associations at the *p* < 0.01 level. In comparing Groups 1 and 2, multivariable models were adjusted for maternal age, smoking, socioeconomic status, plurality, diabetes type, diabetes management, hypertension, previous caesarean section, model of care, mode of delivery and gestational age at birth. Mode of delivery was omitted as a covariate from regression models for the outcome of stillbirth. In comparing Groups 1 and 3, multivariable models were adjusted for maternal age, BMI, region of birth and model of care. Multivariable models were checked for evidence of multicollinearity by examining Pearson correlation coefficients, variance inflation factors and eigenvalues. All analyses were performed using SAS 9.4 (SAS Institute, Cary, NC). Ethics approval, including a waiver of consent, was obtained from the Northern Sydney Local Health District Human Research Ethics Committee (2020/ETH01780).

## 3 Results

**FIGURE 1:**
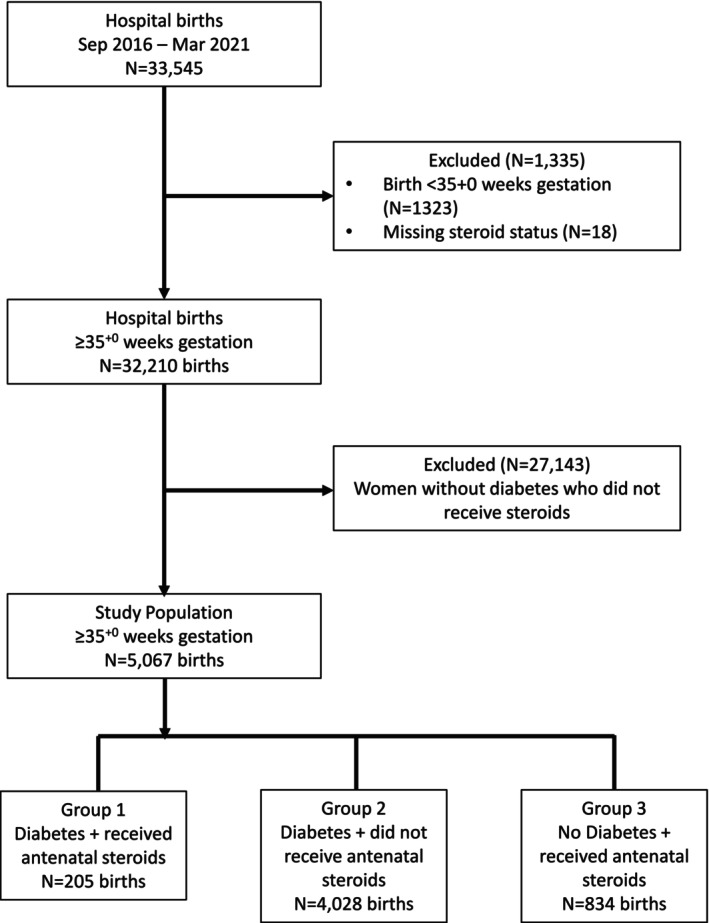
Flowchart of the study population.

Of 5 067 women included in the study, 205 (4.0%) women had diabetes and received antenatal corticosteroids [Group 1], 4 028 (79.5%) had diabetes and did not receive antenatal corticosteroids [Group 2], and 834 (16.5%) did not have diabetes and received antenatal corticosteroids [Group 3] (Figure 1).

### 3.1 Association Between Antenatal Corticosteroids and Study Outcomes

Among women with diabetes, those who received antenatal corticosteroids were more likely to be older, smoke, have a multiple birth, have pre‐existing diabetes and hypertension, have their diabetes managed with insulin, have a previous caesarean section and deliver by caesarean in the current pregnancy, have a hospital‐based medical model of care, and give birth at late preterm to early term gestational ages than women who did not receive corticosteroids (Table 1; Group 1 vs. Group 2).

**TABLE 1:** Characteristics of the study population by comparison group.

Characteristics	Group 1 diabetes + corticosteroids	Group 2 diabetes + no corticosteroids	Group 3 no diabetes + corticosteroids	Group 1 vs. 2 *p*	Group 1 vs. 3 *p*
	*N = 205*	*N = 4 028*	*N = 834*		
Maternal age					
*Mean (sd)*	34.3 (5.7)	33.5 (4.9)	31.9 (5.7)	0.017	< 0.001
< 20 years	^	11 (0.3)	11 (1.3)	0.008	< 0.001
20–24 years	^	157 (3.9)	70 (8.4)		
25–29 years	36 (17.6)	647 (16.1)	197 (23.6)		
30–34 years	73 (35.6)	1 516 (37.6)	269 (32.3)		
35–39 years	55 (26.8)	1 259 (31.3)	219 (26.3)		
40–44 years	29 (14.2)	397 (9.9)	58 (7.0)		
≥ 45 years	7 (3.4)	41 (1.0)	10 (1.2)		
Body Mass Index (kg/m^2^)					
*Mean (sd)*	27.8 (6.6)	27.0 (6.7)	25.1 (6.0)	0.081	< 0.001
Overweight (25.0–29.9)	44 (21.5)	1 062 (26.4)	182 (21.8)	0.053	< 0.001
Obese (≥ 30)	68 (33.2)	1 076 (26.7)	137 (16.4)		
Maternal region of birth					
ANZ + Oceania	118 (57.8)	1 893 (47.1)	603 (72.4)	0.025	< 0.001
South‐East Asia	12 (5.9)	360 (9.0)	38 (4.6)		
North‐East Asia	25 (12.3)	543 (13.5)	44 (5.3)		
South Asia	31 (15.2)	656 (16.3)	42 (5.0)		
Other	18 (8.8)	568 (14.1)	106 (12.7)		
Maternal smoking	27 (13.2)	284 (7.1)	112 (13.4)	0.001	0.922
Socioeconomic status (quintiles)	*N* = 205	*N* = 4024	*N* = 834		
1 (most disadvantaged)	8 (3.9)	118 (2.9)	43 (5.2)	0.003	0.873
2	43 (21.0)	535 (13.3)	180 (21.6)		
3	24 (11.7)	394 (9.8)	111 (13.3)		
4	47 (22.9)	850 (21.1)	177 (21.2)		
5 (least disadvantaged)	83 (40.5)	2 127 (52.9)	323 (38.7)		
Remoteness	*N* = 205	*N* = 4 024	*N* = 834		
Major cities	202 (98.5)	3 966 (98.6)	811 (97.2)	1.000	0.452
Parity					
0	75 (36.6)	1 855 (46.1)	328 (39.3)	0.049	0.199
1	83 (40.5)	1 450 (36.0)	277 (33.2)		
2	31 (15.1)	500 (12.4)	137 (16.4)		
≥ 3	16 (7.8)	223 (5.5)	92 (11.0)		
Plurality					
Singleton	156 (76.1)	3 954 (98.2)	641 (76.9)	< 0.001	0.817
Multiple	49 (23.9)	74 (1.8)	193 (23.1)		
Type of diabetes					
Gestational diabetes	178 (86.8)	3 862 (95.9)	—	< 0.001	—
Type I	18 (8.8)	87 (2.2)	—		
Type II	9 (4.4)	79 (2.0)	—		
Diabetes management					
Diet only	86 (42.0)	1 946 (48.3)	—	0.010	—
Oral hypoglycaemics	7 (3.4)	277 (6.9)	—		
Insulin	112 (54.6)	1 805 (44.8)	—		
Any hypertension	31 (15.1)	249 (6.2)	97 (11.6)	< 0.001	0.173
Previous caesarean section^a^	64 (31.2)	698 (17.3)	274 (32.9)	< 0.001	0.655
Model of care^b^					
GP/GP Obstetrician	^	142 (3.5)	35 (4.2)	< 0.001	< 0.001
Midwifery continuity of care	^	394 (9.8)	69 (8.3)		
Hospital‐based Medical	165 (80.5)	2 455 (61.0)	488 (58.5)		
Hospital‐based Midwifery	16 (7.8)	834 (20.7)	150 (18.0)		
Private Obstetrician	17 (8.3)	203 (5.0)	92 (11.0)		
Mode of delivery					
Vaginal	34 (16.6)	1 997 (49.6)	174 (20.9)	< 0.001	0.160
Instrumental	12 (5.9)	505 (12.5)	30 (3.6)		
Caesarean section	159 (77.6)	1 526 (38.9)	630 (75.5)		
No labour	140 (68.3)	900 (22.9)	551 (66.0)		
Labour	19 (9.3)	626 (16.0)	79 (9.5)		
Gestational age at birth (days)					
245–258 (35^+0^–36^+6^)	87 (42.4)	187 (4.6)	381 (45.7)	< 0.001	0.384
259–272 (37^+0^–38^+6^)	109 (53.2)	1 593 (39.6)	403 (48.3)		
≥ 273 (39^+0^)	9 (4.4)	2248 (55.8)	50 (6.0)		
Birthweight					
SGA < 10th centile	12 (6.0)	91 (2.3)	36 (4.3)	< 0.001	0.326
LGA > 90th centile	6 (3.0)	75 (1.9)	8 (1.0)	0.282	0.038
Baby's year of birth					
2016	^	14 (0.4)	^	0.014	0.904
2017	56 (27.3)	787 (19.5)	223 (26.7)		
2018	60 (29.3)	972 (24.1)	247 (29.6)		
2019	38 (18.5)	928 (23.0)	166 (19.9)		
2020	34 (16.6)	876 (21.8)	146 (17.5)		
2021	^	451 (11.2)	^		

*Note:* ^ Values of 5 or less not displayed for privacy reasons. Baby's year of birth values redacted for 2021 to preserve privacy for 2016 values.

Abbreviation: sd, standard deviation.

^a^ Previous caesarean section given as proportion of women with a previous pregnancy.

^b^ Midwifery continuity of care includes midwifery caseload, team and privately practising midwife. Hospital‐based medical includes high‐risk maternity care.

Unadjusted analyses showed increased risk of postpartum haemorrhage, jaundice, hypoglycaemia, respiratory distress and infant admission to special care nursery or neonatal intensive care unit for women with diabetes who had antenatal corticosteroids compared to those who did not receive corticosteroids (Table 2). However, after adjusting for potential confounders, the relative risk estimates for all outcomes were decreased, and no statistically significant associations persisted (Table 2). There were no neonatal deaths among women who had diabetes. There was no evidence of multicollinearity in the multivariable models. Restricting the corticosteroid group to women who completed their course of corticosteroids (Group 1, *n* = 126), unadjusted and adjusted relative risks of a similar magnitude and direction were obtained (Table S1).

**TABLE 2:** Association between antenatal corticosteroid use and study outcomes among women with diabetes mellitus (Group 1 vs. Group 2, *N* = 4 233).

	Group 1 diabetes + corticosteroids *N* = 205	Group 2 diabetes + no corticosteroids	Unadjusted RR (95% CI)	Adjusted RR (95% CI)^a^
Maternal outcomes				
Postpartum haemorrhage (≥ 500 mL total blood loss)	69 (33.7)	1 036 (25.7)	1.31 (1.07–1.60)	1.01 (0.81–1.26)
Primary PPH (≥ 500 mL within 24 h of birth)	61 (29.8)	911 (22.6)	1.32 (1.06–1.64)	1.00 (0.79–1.28)
Severe PPH (≥ 1 000 ml total blood loss)	17 (8.3)	255 (6.3)	1.31 (0.82–2.10)	0.88 (0.52–1.46)
Infection	^	33 (0.8)	0.60 (0.08–4.33)	—
**Infant Outcomes**				
Jaundice	42 (20.5)	432 (10.7)	1.91 (1.44–2.54)	0.87 (0.63–1.21)
Hypoglycaemia	78 (38.1)	897 (22.3)	1.71 (1.42–2.05)	1.04 (0.84–1.27)
Respiratory distress	43 (21.0)	374 (9.3)	2.26 (1.70–3.00)	0.94 (0.69–1.27)
SCN/NICU admission	114 (55.6)	786 (19.5)	2.85 (2.48–3.27)	1.14 (0.98–1.32)
Stillbirth (per 1 000 births)^b^	^	7 (1.7)	2.81 (0.35–22.7)	—

*Note:* Adjusted RRs could not be estimated for infection and stillbirth. ^ Values of 5 or less not displayed for privacy reasons.

^a^ RRs for Group 1 relative to Group 2. Multivariate models adjusted for maternal age, smoking status, socioeconomic status, plurality, diabetes type, diabetes management, hypertension, previous caesarean, model of care, mode of delivery and gestational age at birth. Selection of variables for inclusion in multivariate model based on a priori knowledge and cut‐off of *p* < 0.01 in univariate associations.

^b^ Not adjusted for mode of delivery.

### 3.2 Association Between Diabetes in Pregnancy and Study Outcomes

Among women who received corticosteroids, those who had diabetes were more likely to be older, have a higher body mass index, and have a hospital‐based medical model of care than women who did not have diabetes (Table 1; Group 1 vs. Group 3). Among women who had corticosteroids, there were similar proportions of corticosteroid course completion among women with diabetes (61.5%) and those without diabetes (63.2%). There were also similar proportions of women who had at least one dose of corticosteroids more than seven days prior to birth (diabetes: 19.5% vs. no diabetes: 19.3%). The frequency of incomplete course of corticosteroids less than 24 h before birth was 17.1% among women with diabetes compared to 14.5% among women without diabetes.

There was an increased risk of neonatal hypoglycaemia (aRR 1.61, 95% CI 1.28–2.02) and admission to NICU (aRR 1.18, 95% CI 1.02–1.38) for infants born to women with diabetes, after adjusting for potential confounders (Table 3). There were no neonatal deaths among women who received corticosteroids. In analyses restricted to women who received a complete course of corticosteroids, similar results were observed with statistically significant associations between diabetes and increased risk of neonatal hypoglycaemia (aRR 1.84, 95% CI 1.38–2.46) and NICU admission (aRR 1.30, 95% CI 1.06–1.59) (Table S2).

**TABLE 3:** Association between diabetes status and study outcomes among women who received antenatal corticosteroids (Group 1 vs. Group 3, *N* = 1 039).

	Group 1 diabetes + corticosteroids *N* = 205	Group 3 no diabetes + corticosteroids *N* = 834	Unadjusted RR (95% CI)	Adjusted RR (95% CI)^a^
Maternal outcomes				
Postpartum haemorrhage (≥ 500 mL total blood loss)	69 (33.7)	270 (32.4)	1.04 (0.84–1.29)	0.95 (0.76–1.20)
Primary PPH (≥ 500 mL within 24 h of birth)	61 (29.8)	231 (27.7)	1.07 (0.85–1.36)	0.95 (0.75–1.21)
Severe PPH (≥ 1 000 mL total blood loss)	17 (8.3)	59 (7.1)	1.17 (0.70–1.97)	1.03 (0.60–1.75)
Infection	^	12 (1.4)	0.34 (0.04–2.59)	—
**Infant Outcomes**				
Jaundice	42 (20.5)	154 (18.5)	1.11 (0.82–1.51)	0.97 (0.70–1.34)
Hypoglycaemia	78 (38.1)	203 (24.3)	1.56 (1.26–1.93)	1.61 (1.28–2.02)
Respiratory distress	43 (21.0)	152 (18.2)	1.15 (0.85–1.56)	1.19 (0.86–1.64)
SCN/NICU admission	114 (55.6)	394 (47.2)	1.18 (1.02–1.36)	1.18 (1.02–1.38)
Stillbirth (per 1 000 births)	^	^	2.03 (0.19–22.3)	—

*Note:* Adjusted RRs could not be estimated for infection and stillbirth. ^ Values of 5 or less not displayed for privacy reasons.

^a^ RRs for Group 1 relative to Group 3. Multivariate models adjusted for maternal age, body mass index, maternal region of birth and model of care. Selection of variables for inclusion in multivariate model based on a priori knowledge and cut‐off of *p* < 0.01 in univariate associations.

## 4 Discussion

This population‐based cohort study found that there were no differences in maternal and neonatal outcomes for women who had diabetes and received corticosteroids compared to those who had diabetes and did not receive corticosteroids, including no difference in rates of neonatal respiratory distress. While the two groups were different in relation to gestational age at birth and infant outcomes, particularly those related to gestational age such as jaundice, hypoglycaemia, respiratory distress and transfer to higher care, these differences disappear after adjustment. This is an interesting finding considering antenatal corticosteroids are administered to reduce respiratory‐related morbidity and mortality in preterm infants. We also found that women who had diabetes and received corticosteroids had a higher risk of having an infant with neonatal hypoglycaemia or requiring NICU/SCN admission compared to women without diabetes who received corticosteroids.

Our findings of no benefit of antenatal corticosteroids for babies born to women with diabetes in the late preterm period are consistent with other cohort studies in the literature [17, 18]. A US multicentre cohort study found no difference in hypoglycaemia and respiratory morbidity associated with corticosteroid exposure in women with gestational and pre‐gestational diabetes; however, they also reported decreased use of supplemental oxygen, mechanical ventilation and respiratory distress with two doses of antenatal corticosteroids [17]. Similarly, a cohort study in Turkey found there was no difference in composite neonatal or composite respiratory morbidity outcomes associated with corticosteroid use in women with gestational diabetes [18]. A scoping review also found there was no clear evidence of benefit of antenatal corticosteroids for women with diabetes on neonatal respiratory outcomes [19]. More worryingly, a recent large population‐based United States study found an increased risk of adverse neonatal composite outcomes for women with GDM who were given antenatal corticosteroids compared to women with GDM without corticosteroids, which was mostly driven by increased rates of adverse respiratory outcomes (immediate assisted ventilation, intubation > 6 h and surfactant use) for those babies receiving corticosteroids [20]. Furthermore, a population‐based US study reported worse neonatal respiratory outcomes associated with antenatal corticosteroid use in late preterm births complicated by pre‐gestational diabetes than those who did not receive corticosteroids. Specifically, they reported higher adjusted odds for assisted ventilation, NICU admission and surfactant use [21].

Our study also found that women who had diabetes and received corticosteroids had a higher risk of having an infant with hypoglycaemia and their infant requiring NICU/SCN admission compared to women without diabetes who received corticosteroids, consistent with the higher risk profile of infants of mothers with diabetes. Neonatal hypoglycaemia is a well‐established and preventable cause of neonatal brain injury, and its definition (glucose concentration < 2.6 mmol/L) is based on the association found between this level of glucose concentration and increased risk of developmental delay among preterm babies at 18 months corrected age [22]. Furthermore, there is evidence of long‐lasting adverse effects of neonatal hypoglycaemia on neurodevelopment in later childhood [23]. Maternal hyperglycaemia has also been associated with respiratory distress via the mechanism of decreased surfactant production [24, 25]. Corticosteroid administration increases surfactant production but also worsens maternal hyperglycaemia, and it is not known what the overall benefit/harm is. Previous randomised controlled trials assessing the benefits of antenatal corticosteroids for fetal lung maturation found an increase in neonatal hypoglycaemia, but many of these studies excluded women with gestational and/or pre‐existing diabetes [4]. The landmark Antenatal Late Preterm Steroids (ALPS) trial, which included women with gestational diabetes, found a significant proportion of neonates developed hypoglycaemia (22%) compared to controls (13.6%) [5].

Emerging evidence from a systematic review suggests that exposure to a course of antenatal corticosteroids is associated with increased risk of adverse neurocognitive and psychological outcomes in children born at late preterm and full‐term gestations [26]. However, a follow‐up study of the ALPS cohort, which included infants who experienced hypoglycaemia, found no difference in neurodevelopmental outcomes at 6 years of age or older between children born to women who received antenatal corticosteroids compared to those who did not [27]. All of these findings, taken together with no evidence of short‐term benefit of antenatal corticosteroids demonstrated by our study and others, suggest questionable utility and benefit of administering corticosteroids in this group. Accordingly, the American College of Obstetrics and Gynaecology does not recommend antenatal corticosteroid use in women with diabetes in the late preterm period, noting that women with pre‐existing diabetes were excluded from the ALPS trial [5, 28]. The Royal College of Obstetrics and Gynaecology does not routinely recommend corticosteroid administration for women with diabetes in the late preterm setting and highlights the evidence of increased neonatal hypoglycaemia [29]. In contrast, the WHO guidelines state that fetal lung immaturity is more likely in women with diabetes and support antenatal corticosteroid administration > 34 weeks, especially if maternal blood sugars are poorly controlled [30].

The strengths of this study include the prospectively collected and detailed nature of the clinical data reflecting contemporary practice. Furthermore, the same diagnostic thresholds for diabetes were applied throughout the study period in one of the tertiary facilities contributing up to half of the births studied, meaning less heterogeneity within the group of women with diabetes. However, the use of routinely collected data meant that important information was sometimes unavailable. It is unknown why some women with diabetes were prescribed antenatal corticosteroids while others were not. Data on glycaemic control during pregnancy were unavailable, and as a result we could not account for its effects on the study outcomes. Also, it was unknown if insulin or other medications were used to manage maternal hyperglycaemia and subsequent neonatal hypoglycaemia in the corticosteroid groups. While there was adjustment for differences between groups, we acknowledge that the group receiving antenatal corticosteroids was fundamentally different from the group that did not receive antenatal corticosteroids and residual confounding remains despite statistical adjustment. There may have been ascertainment bias for neonatal hypoglycaemia as babies delivered at term without other risk factors (such as maternal diabetes) are not routinely tested for hypoglycaemia; however, we did adjust for gestational age at birth to account for the difference in gestational age distribution between groups 1 and 2. A further limitation of this study is that it did not include the full range of late preterm births (i.e., 34^+0^–36^+6^ weeks), which may underrepresent the benefit of antenatal corticosteroids if the benefits vary with gestational age. This was a large study with an ethnically diverse population; however, the findings may not be generalisable to facilities with different practices.

In conclusion, these findings underscore the importance of robust evidence to guide treatment plans and to understand which patients may benefit from antenatal corticosteroids. Clinicians should weigh the benefits of antenatal corticosteroids in improving fetal lung maturity against the potential for increased neonatal complications, particularly in late preterm to early term deliveries. There is a clear need for a multicentre randomised controlled trial that investigates the benefits and harms of antenatal corticosteroids in women with diabetes (pre‐existing and gestational) in the late preterm period.

## Funding

This work was supported by the National Health and Medical Research Council (Grant No. 1186572), the NSW Ministry of Health, the Royal North Shore Hospital No. 2 Trust Fund Research Registrar Funding, the Australian Diabetes Society, and the Douglas and Lola Douglas Scholarship.

## Conflicts of Interest

The authors declare no conflicts of interest.

## Supporting information


**Table S1:** Association between antenatal corticosteroid use (completed course) and study outcomes among women with diabetes mellitus (Group 1 vs. Group 2, *N* = 4 154).
**Table S2:** Association between diabetes status and study outcomes among women who received a complete course of antenatal corticosteroids (Group 1 vs. Group 3, N = 653).

## Data Availability

The data that support the findings of this study cannot be shared publicly for privacy reasons. Approvals granted for the use of these data do not permit sharing of the data. Data can be obtained upon gaining appropriate approvals and clearances and upon request to the New South Wales Ministry of Health.
