# Supplementary material for: Outcomes for Women With Diabetes at Late Preterm Gestation Who Received Antenatal Corticosteroids: A Population‐Based Cohort Study
Source: Aust N Z J Obstet Gynaecol. 2026 Jul 28;66(4):e70164. doi: 10.1111/ajo.70164 (PMC13416006; doi:10.1111/ajo.70164)
Supplement: Supplementary file 1 — Table S1: Association between antenatal corticosteroid use (completed course) and study outcomes among women with diabetes mellitus (Group 1 vs. Group 2, N = 4 154). Table S2: Association between diabetes status and study outcomes among women who received a complete course of antenatal corticosteroids (Group 1 vs. Group 3, N = 653). [file AJO-66-0-s001.docx]

**SUPPLEMENTARY MATERIALS**

**Table S1. Association between antenatal corticosteroid use (completed course) and study outcomes among women with diabetes mellitus (Group 1 vs Group 2, N=4,154)**

|  | **Group 1 Diabetes + Corticosteroids**  **N=126** | **Group 2**  **Diabetes + No Corticosteroids**  **N=4,028** | **Unadjusted**  **RR (95% CI)** | **Adjusted RR**  **(95% CI)*** |
| --- | --- | --- | --- | --- |
| **Maternal Outcomes** |  |  |  |  |
| Postpartum haemorrhage (≥500ml total blood loss) | 49 (38.9) | 1036 (25.7) | 1.51 (1.21-1.89) | 1.11 (0.86-1.44) |
| Primary PPH  ((≥500ml within 24hrs of birth) | 45 (35.7) | 911 (22.6) | 1.58 (1.24-2.01) | 1.15 (0.87-1.52) |
| Severe PPH  (≥1000ml total blood loss) | 13 (10.3) | 255 (6.3) | 1.63 (0.96-2.76) | 0.97 (0.55-1.71) |
| Infection | ^ | 33 (0.8) | 0.97 (0.13-7.03) | - |
| **Infant Outcomes** |  |  |  |  |
| Jaundice | 28 (22.2) | 432 (10.7) | 2.07 (1.48-2.91) | 0.90 (0.62-1.33) |
| Hypoglycaemia | 51 (40.5) | 897 (22.3) | 1.82 (1.46-2.26) | 1.02 (0.81-1.30) |
| Respiratory distress | 27 (21.4) | 374 (9.3) | 2.31 (1.63-3.27) | 0.92 (0.64-1.32) |
| SCN/NICU admission | 71 (56.4) | 786 (19.5) | 2.89 (2.45-3.41) | 1.11 (0.93-1.32) |
| Stillbirth (per 1000 births)** | 0 (0.0) | 7 (1.7) | - | - |

* RRs for Group 1 relative to Group 2. Multivariate models adjusted for maternal age, smoking status, socioeconomic status, plurality, diabetes type, diabetes management, hypertension, previous caesarean, model of care, mode of delivery and gestational age at birth. Selection of variables for inclusion in multivariate model based on a priori knowledge and cut-off of p<0.01 in univariate associations.

**not adjusted for mode of delivery

-RRs could not be estimated

^ Values of 5 or less not displayed for privacy reasons

**Table S2. Association between diabetes status and study outcomes among women who received a complete course of antenatal corticosteroids (Group 1 vs Group 3, N=653)**

|  | **Group 1 Diabetes + Corticosteroids**  **N=126** | **Group 3**  **No diabetes + corticosteroids**  **N=527** | **Unadjusted**  **RR (95% CI)** | **Adjusted RR**  **(95% CI)*** |
| --- | --- | --- | --- | --- |
| **Maternal Outcomes** |  |  |  |  |
| Postpartum haemorrhage (≥500ml total blood loss) | 49 (38.9) | 168 (31.9) | 1.22 (0.95-1.57) | 1.09 (0.83-1.43) |
| Primary PPH  ((≥500ml within 24hrs of birth) | 45 (35.7) | 140 (26.6) | 1.34 (1.02-1.77) | 1.15 (0.86-1.55) |
| Severe PPH  (≥1000ml total blood loss) | 13 (10.3) | 42 (8.0) | 1.29 (0.71-2.34) | 1.09 (0.59-2.04) |
| Infection | ^ | 7 (1.3) | 0.60 (0.07-4.81) | - |
| **Infant Outcomes** |  |  |  |  |
| Jaundice | 28 (22.2) | 105 (19.9) | 1.12 (0.77-1.62) | 0.94 (0.63-1.41) |
| Hypoglycaemia | 51 (40.5) | 126 (23.9) | 1.69 (1.30-2.20) | 1.84 (1.38-2.46) |
| Respiratory distress | 27 (21.4) | 82 (15.6) | 1.38 (0.93-2.03) | 1.44 (0.95-2.18) |
| SCN/NICU admission | 71 (56.4) | 227 (43.1) | 1.31 (1.09-1.57) | 1.30 (1.06-1.59) |
| Stillbirth (per 1000 births) | 0 (0.0) | ^ | - | - |

*RRs for Group 1 relative to Group 3. Multivariate models adjusted for maternal age, body mass index, maternal region of birth and model of care. Selection of variables for inclusion in multivariate model based on a priori knowledge and cut-off of p<0.01 in univariate associations.

-RRs could not be estimated

^ Values of 5 or less not displayed for privacy reasons
